# Using chimeric antigen receptor T-cell therapy to fight glioblastoma multiforme: past, present and future developments

**DOI:** 10.1007/s11060-021-03902-8

**Published:** 2021-11-26

**Authors:** David C. Soler, Amber Kerstetter-Fogle, Thomas S. McCormick, Andrew E. Sloan

**Affiliations:** 1 Department of Neurosurgery, Case Western Reserve University School of Medicine, Cleveland, OH 44106 USA; 2 The Brain Tumor and Neuro-Oncology Center, Cleveland, OH 44106 USA; 3 Department of Dermatology, Cleveland, OH 44106 USA; 4 The Murdough Family Center for Psoriasis, Cleveland, OH 44106 USA; 5 Department of Neurological Surgery, University Hospitals-Cleveland Medical Center and the Case Comprehensive Cancer Center, 11000 Euclid Avenue, Cleveland, OH 44106 USA

**Keywords:** CAR-T, GBM, Novel therapies

## Abstract

**Introduction:**

Glioblastoma multiforme (GBM) constitutes one of the deadliest tumors to afflict humans, although it is still considered an orphan disease. Despite testing multiple new and innovative therapies in ongoing clinical trials, the median survival for this type of malignancy is less than two years after initial diagnosis, regardless of therapy. One class of promising new therapies are chimeric antigen receptor T cells or CAR-T which have been shown to be very effective at treating refractory liquid tumors such as B-cell malignancies. However, CAR-T effectivity against solid tumors such as GBM has been limited thus far.

**Methods:**

A Pubmed, Google Scholar, Directory of Open Access Journals, and Web of Science literature search using the terms chimeric antigen receptor or CAR-T, GBM, solid tumor immunotherapy, immunotherapy, and CAR-T combination was performed for publication dates between January 1987 and November 2021.

**Results:**

In the current review, we present a comprehensive list of CAR-T cells developed to treat GBM, we describe new possible T-cell engineering strategies against GBM while presenting a short introductory history to the reader regarding the origin(s) of this cutting-edge therapy. We have also compiled a unique list of anti-GBM CAR-Ts with their specific protein sequences and their functions as well as an inventory of clinical trials involving CAR-T and GBM.

**Conclusions:**

The aim of this review is to introduce the reader to the field of T-cell engineering using CAR-Ts to treat GBM and describe the obstacles that may need to be addressed in order to significantly delay the relentless growth of GBM.

**Supplementary Information:**

The online version contains supplementary material available at 10.1007/s11060-021-03902-8.

## Introduction

Glioblastoma multiforme (GBM) is the most common primary malignant brain tumor affecting up to 17 individuals per 500,000 adults per year. Despite decades of research, the prognosis is dismal with a median survival of less than 15 months with standard of care [1]. Current standard-of-care treatment regimens consist of tumor de-bulking followed by concomitant chemotherapy and radiation. Recent research has focused on therapies targeting the immune microenvironment of the tumors, as progression of GBM occurs concomitantly with high levels of immunosuppression. Many therapeutic strategies that have been successful with other cancers have failed in GBM as a result of its unique organ localization and immunosuppressive environment [2]. The three main immunotherapies used against GBM to date have been immune checkpoint inhibition, vaccination, and adoptive transfer of effector lymphocytes, with varying outcomes but none has consistently extended survival beyond 12 months. More recently, a newer immunotherapy using oncolytic viruses has shown significant improvement of mean survival [3]

One of the main obstacles conventional immunotherapies for GBM face is the low abundance of leukocytes in the brain under steady-state conditions. Although immune surveillance cells such as T cells and microglia exist, they are located in the choroid plexus stroma and the cerebrospinal fluid (CSF), which occupy the perivascular spaces. The central nervous system (CNS) has traditionally been regarded as immune privileged, and therefore excluded from the protection systemic immune surveillance affords other organs [4]. A distinct obstacle facing drugs and general oncolytic treatments is the presence of the blood brain barrier (BBB) or the blood-cerebrospinal fluid barrier that effectively blocks their entrance into the brain parenchyma where GBM-associated tumors are located. The absence of lymphatic vessels which normally drain antigen presenting cells further confirm the notion of the brain as an immune privileged site.

Single cell sequencing and flow cytometry has clarified the complexity of the cells that reside in the brain parenchyma. The most prominent immune cells in the brain are microglial cells, which serve as the first line of defense against pathogens [5]. Bone marrow-derived macrophages/monocytes are the primary immune cell in glioma and they may compose up to 30% of tumor mass. As such, there are two distinct populations: glioma bone marrow-derived macrophages and microglia.

During tumor progression, monocytes and T cells extravasate into the microenvironment through the compromised BBB. One of the most prominent T cell types in glioblastoma are CD8^+^ cells [6, 7]. These CD8^+^ cytotoxic T cells increase in glioblastoma due to an upsurge in chemoattractants such as CXCL9, CXCL10, and adhesion molecules such as ICAM. However, this massive infiltration eventually creates a heavily immunosuppressive tumor micro-environment (TME) by activation of tumor-associated macrophages (TAMs) and further recruitment of myeloid derived suppressor cells (MDSC) [8]. MDSCs can suppress cytotoxic CD8^+^ T cell proliferation and overall activation by—among others- increasing surface expression of IL-4Rα and the production of arginase and inducible nitric oxide synthase (iNOS) [9]. In response to inflammatory stimuli, such as tumor growth, brain stromal cells produce high levels of classic immunosuppressive cytokines such as transforming growth factor beta (TGF-β) and interleukin-10 (IL-10), which counteract the inflammatory cytokine signals to maintain homeostasis. Glioma cells also are known to produce huge amounts of indolamine 2,3 dioxygenase (IDO) which results in accumulation of regulatory T cells (Treg) which further suppress cytotoxic T cell activity. Combination therapy of patients with brain tumors has been focused on inhibiting the specific immunosuppressive factors, but targeting TGF-β and IDO has shown no clinical benefit so far, despite its success in animal models [10].

The ongoing success in the treatment of many other types of cancers and further progress in understanding of T cell immunotherapy, suggests further novel treatment strategies may be forthcoming for the treatment of brain cancers [11], including the up-and-coming new therapy of Chimeric Antigen Receptor T cells (CAR-Ts).

### Introduction and brief history of CAR-T

Chimeric antigen receptor T cells or CAR-Ts are synthetic immune receptors that redirect cytotoxic T cells to specific targets through recognition of surface proteins expressed on targeted tumor cells. Thus, the principle of CAR-Ts is to genetically modify [12] existing T-cells from cancer patients in order to re-direct their immune response machinery toward a malignant target cell of interest. Theoretically, any cell surface molecule can be targeted through a CAR-T, thus over-riding the tolerance to self-antigens and the antigen recognition gaps in the T- cell repertoire that often limit their scope of reactivity. It should be noted that expression of CAR-Ts basically bypasses the HLA-restricted nature of T-cells, making them immune to evasion strategies such as MHC shedding by tumors. However, the CAR-T needs to be optimized in order to increase its binding and signaling properties. Interestingly, the persistence and strength of CAR-Ts can also be modulated by the design of the intracellular signaling domains.

**Fig. 1:**
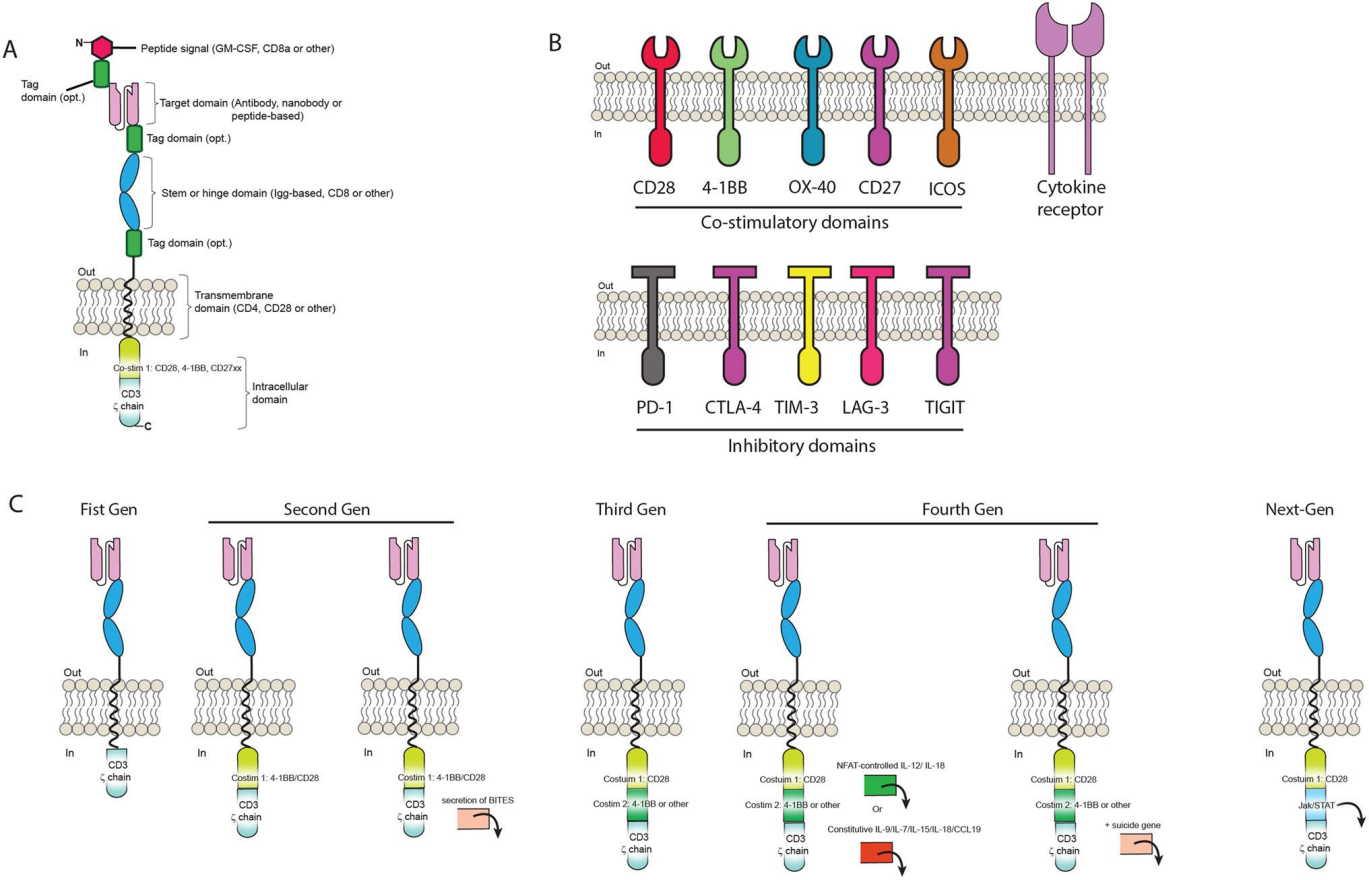
CAR-T design and current generations. **A** The basic CAR-T architecture consists of a target domain followed by a stem or hinge, a transmembrane domain and intracellular co-stimulatory motifs. Tags for easy identification can be placed in different key locations as shown. **B** Several types of co-stimulatory domain targets have been characterized to be used with CAR-T. **C** CAR-T have experienced a fast evolution, from the initial First Generation (First Gen) comprising only one stimulatory domain, all the way to second, third, fourth and next-generation CARs, comprising several combinations of co-stimulatory domain molecules as well as JAK/STAT signaling (Next-Gen)

CAR-Ts have three main domains: the extracellular domain, which includes the antigen recognition domain, a transmembrane domain, and the intracellular domain, which is important for signal transmission (Fig. 1A). CAR-Ts are able to recognize antigen on any HLA background and can target tumor cells that have downregulated HLA expression or proteosomal antigen processing, mechanisms often attributed to tumor escape. A crude version of CAR-Ts was initially reported in 1987 by Yoshihisa Kuwana et al., [13] and almost concomitantly by Gideon Gross and Zelig Eshhar in 1989 [14]. While the first generation CAR-Ts were developed based on work from the Weiss lab demonstrating that inclusion of a CD3ζ domain could activate T-cells [15] (Fig. 1A), more versions followed quickly. Margo Roberts and Finney and colleagues during the 1990s contributed to the development of second generation CAR-Ts by incorporating co-stimulatory domains [16] such as CD28 which improved IL-2 production in Jurkat cells by 20-fold [17] (Fig. 1B). One of the advantages to CAR-Ts is their modular design properties that provide flexible domains which can be adjusted or swapped. Another seminal clinical improvement was the key finding of Rosenberg et al. in 1988, who demonstrated that that mild lympho-depletion in treated patients improved the proliferation of infused tumor-infiltrating lymphocytes (TILs) [18]. However, lympho-depletion is accompanied by very severe side-effects. All these crucial developments, and many others [19], paved the way for the eventual success of CAR-T cells clinically.

However, unequivocal CAR-T results in a clinical setting were not demonstrated until two groups, one lead by Drs. June and Levine and funded by the Alliance for Cancer Gene Therapy or ACGT, and another by Rosenberg et al., from the NIH who showed remission in B-cell leukemia and lymphoma patients using a CAR-T approach [20, 21]. After this initial success combined with a little luck [22], more clinical trials followed [19, 23]. Experience with CD19-targeted CAR-Ts have had remarkable outcomes for patients with CD19-positive B cell malignancies which lead to FDA approval in 2017 [24, 25]. These CD19-targeted-CAR T therapies and the adoptive cellular therapy that followed in melanoma have also raised optimism for the treatment of CNS malignant tumors. For example, in patients with metastatic melanoma, Hong reported that 35% of patients achieved complete response in the brain metastases as well as extra-cranial disease [26].

As a result of the success of CAR-T cells on refractory liquid tumors, CAR-T technology has expanded substantially in recent years. Tagging the CAR-T at its amino (N)-terminus, upper or lower stem without losing functionality (Fig. 1A) has also been described when availability for detection is difficult [27]. Also of importance is the length of the CAR-T hinge [28], which ultimately can make a CAR-Ts effective in vitro but not in vivo [29]. This phenomenon is very unfortunate, because the vice-versa effect could also happen, i.e., where CAR-Ts don’t work in vitro but may work in vivo. Unfortunately such CAR-Ts would never be developed because very seldom do in vitro failures lead to *in* vivo testing.

Given the clinical success of CAR-Ts, development of co-stimulatory domains has been extensively characterized [30] as shown in Fig. 1B, as well as using nanobodies instead of antibodies as the targeting domain [31]. Nanobodies are smaller than conventional antibodies and are being explored in CAR-Ts in order to gain accessibility to difficult antigens [32]. Owing to the flexible nature of proteins, many more versions have been generated including third, fourth and next-generation CAR-Ts by incorporating JAK/Stat signaling (Fig. 1C).

**Fig. 2:**
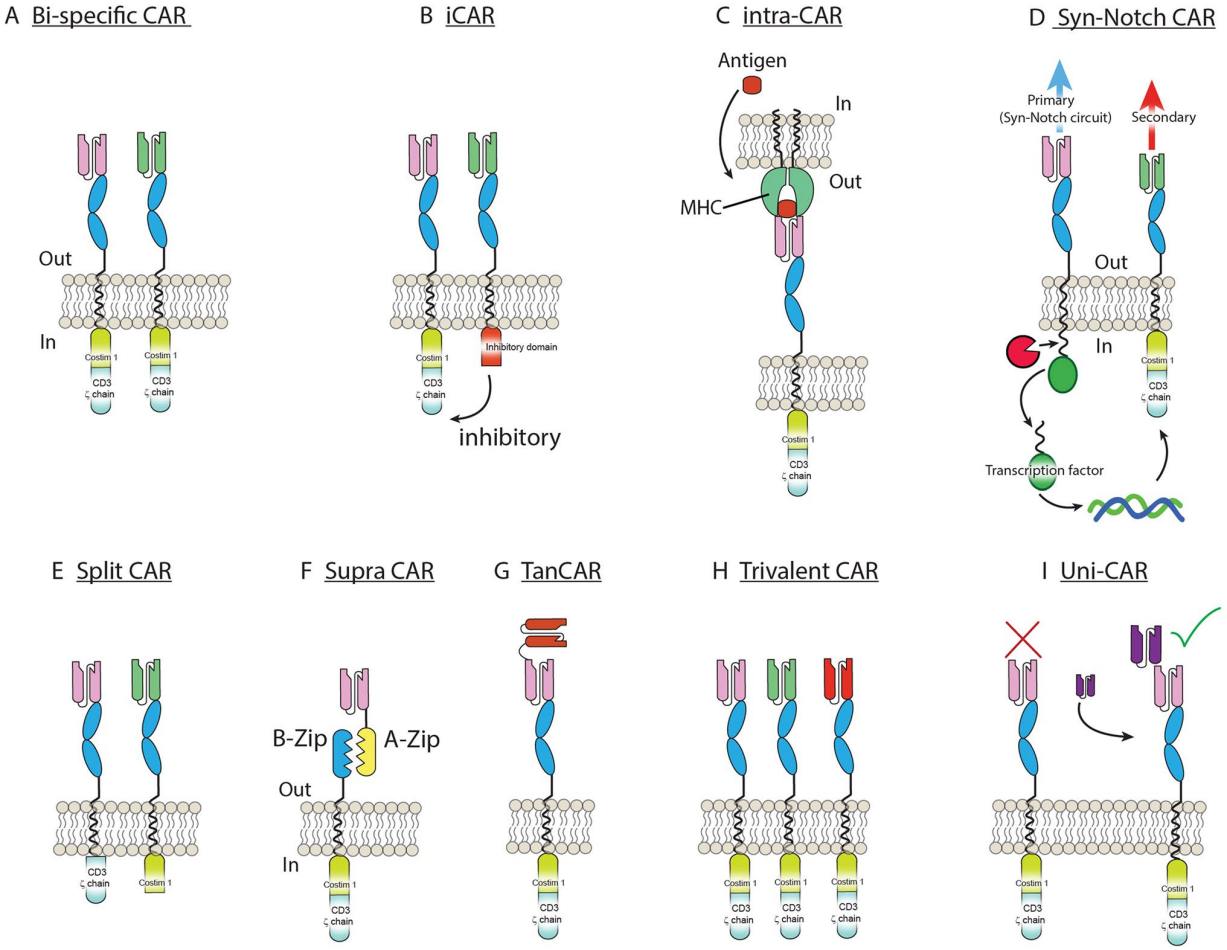
Types of CAR-T developed. Several different strategies have been developed involving CAR-Ts owing to their flexible nature. **A** Bi-specific CAR, targeting two antigens independently. **B** iCAR, targeting two independent antigens leads to inhibition (thus sparing healthy tissues). **C** Intra-CAR-T can be designed to target antigens that are located intracellularly but expressed via MHC. **D** Syn-Notch CAR-T where a secondary-targeting antigen is strongly controlled by binding of a primary one. **E** Split CAR-T are created by targeting two independent antigens with the co-stimulatory domains split. Thus, only when both antigens are present is the CAR-T fully activated. **F** Supra-CAR: the CAR-T construct is split allowing for one co-stimulatory domain (B-zip) to bind several targeting domains (A-zip). **G** Tan-CAR-T are achieved by fusing two antigen-targeting domains into one. **H** Trivalent CAR-T, where three independent antigen-targeting CAR-T are expressed independently within the same cell. **I** Uni-CAR-T consist of an antigen-binding domain that bind not an endogenous target but a soluble one administered exogenously acting as a bridge (purple)

While liquid tumors such as B-cell malignancies have experienced breathtaking success in achieving remission in up to 70–90% of treatment-refractory cancers, CAR-Ts designed to treat solid tumors have had considerably less success so far. Solid tumors pose greater challenges than liquid given the existence of a complex and heavily immuno-suppressed TME that surrounds the tumor and protects it from destruction [33]. The TME may also hamper the T-cell trafficking and infiltration necessary, resulting in T-cell exhaustion [34], and CAR-Ts that address the TME problem display enhanced anti-tumor efficacy in vivo [35]. Further, in some solid tumors, such as glioma, the TME is characterized by low nutrient regions and hypoxia [36]. However, given the versatile nature that CAR-T cell engineering offers, several improvements can be made to CAR-Ts in order to address the unique challenges that solid tumors present (Fig. 2). Other approaches to minimize TME effects have been; (1) designing CAR-Ts with dominant-negative TGFβrII receptors –dnTGFβII-, expression of mutant forms of FAS [37], (2) PD-1-CAR-T [38], dominant-negative PD-1 [39] or 3) as we describe below-secretion of key cytokines or heparanase [40].

## Methods

Electronic databases, Pubmed, Google Scholar, Directory of Open Access Journals, and Web of Science, were searched from January 1987 to August 2021. Database searches included the following key words: ‘glioblastoma or glioma’, ‘solid tumor immunotherapy’, ‘CAR-T’, ‘immunotherapy’, and ‘CAR-T combination’. We further manually screened references within the related articles to expand the search range. Two researchers (AKF and DS) extracted the relevant information and validated their inclusion in the current review.

### CAR-T versus GBM

**Fig. 3:**
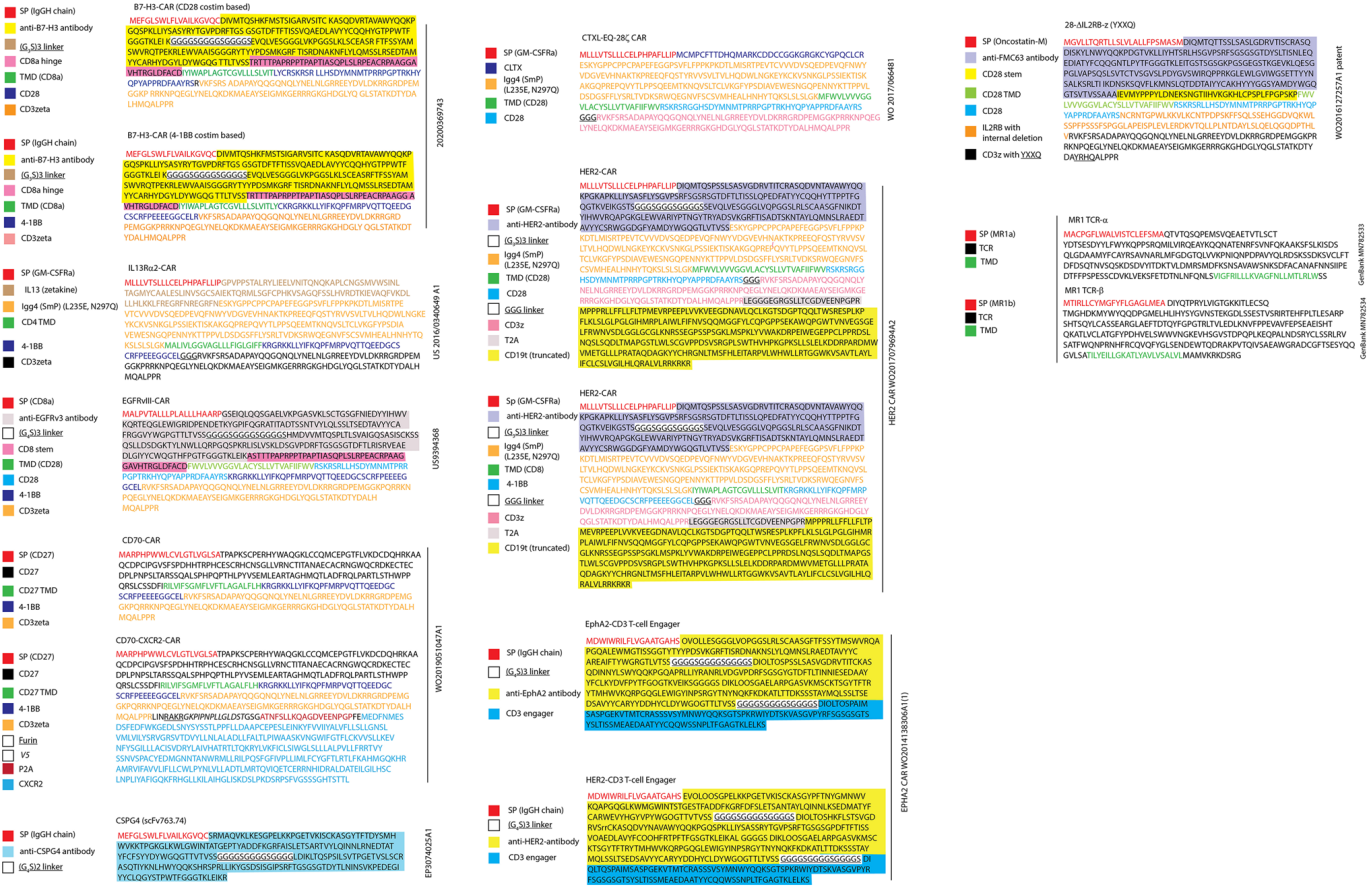
List of anti-GBM CAR-T amino acid sequences. A comprehensive list of several CAR-T targeting different antigens on GBM is shown based on an extensive literature search. Within each CAR-T, different colors on amino acid sequences identify their nature and functionality. *TMD* transmembrane domain, *CLTX* chlorotoxin peptide

Several CAR-Ts have been developed to treat GBM and other solid tumors, but to-date none has led to long-term remission [11]. Despite these initial failures, the use of CAR-Ts against GBM is ongoing with multiple designs undergoing clinical trials (Table 1). The flexibility that T-cell genetic engineering offers is a very strong impetus to improve tumor targeting in a way no other treatment allows: i.e., using the plethora of cell-killing mechanisms that the immune system offers. CAR-Ts have been successfully used to treat several liquid (blood) cancers owing to the existence of very specific tumor targets. Thus, the rationale behind using CAR-Ts to treat GBM is that sooner or later a way to target multiple highly-specific tumor targets will be discovered. Indeed, multiple GBM-specific targets have already been identified, the most notable including EGFRvIII [41–43], HER2 [44], EphA2 [45] or IL-13Rα2-CAR [46, 47] among many others identified in Fig. 3 and Supplementary Table 1. Novel designs include an IL-13Rα2-CAR developed by Brown et al. [48], using a peptide as the targeting-domain (zetakine) or by Pituch et al., using an antibody [47]. Both approaches showed promise in murine studies targeting the IL-13Rα2 receptor in glioma cells and the zetakine version induced remission in a Phase I human GBM trial [46]. However, in this clinical trial the remission was relatively short-lived, since under the IL-13Rα2-CAR pressure, the tumor is suspected to have undergone antigenic loss, an acknowledged major obstacle in combating GBM with CAR-T therapy, since it represents the disappearance of the primary CAR-T target, which renders CAR-Ts ineffective. To compound this problem, antigenic loss can be the result of the heterogeneous cellular nature of GBM, with existence of GBM IL-13Rα2^neg^ cells that expand to bypass CAR-T or instead IL-13Rα2^+^ cells that somehow undergo gene loss of IL-13Rα2. However, a clinical trial conducted by Brown et al., demonstrated that despite a heavily immunosuppressive milieu, CAR-Ts were able to eradicate IL-13Rα2^+^ cells. More recently, a T cell receptor fusion construct (TRuC) against IL-13Rα2 has been developed and tested in a U251 NG1 murine model of GBM, showing superior reactivity and safety profiles compared to conventional CAR-T cells [49].

**Table 1:** List of clinical trials involving CAR-T and GBM

Molecular Target	CAR T-cell therapy	Phase	Estimated enrollment	Clinical trial reference number	CAR-T cell dosage	Status	Location	Estimated primary completion date
B7-H3	Pilot study of B7-H3 CAR-T in treating patients with recurrent and refractory glioblastoma	1	12	NCT04385173	Three intratumoral or intracerebroventricular injectionsof CAR-T at two doses in between temozolomide cycles	Recruiting	Second Affiliated Hospital, School of Medicine, Zhejiang University, China	May, 2022
	B7-H3 CAR-T for recurrent or refractory glioblastoma	1/2	40	NCT04077866	Three intratumoral or intracerebroventricular injectionsof CAR-T at two doses in between temozolomide cycles	Recruiting	Second Affiliated Hospital, School of Medicine, Zhejiang University, China	June, 2024
Chlorotoxin (CLTX)	Chimeric antigen receptor (CAR) T cells with a cholrotoxin tumor-targeting domain for the treatment of MPP2^+^ recurrent or progressive glioblastoma. NCT04214392	1	36	NCT04214392	Three weekly cycles of one or two CAR-Tcell infusions	Recruiting	City of Hope Medical Center, CA, United States	February, 2023
HER2	Intracerebral EGFR-vIII CAR-T cells for recurrent GBM	1	24	NCT03283631	Starting doseof 2.5 × 108 CAR-T cellsper intracerebral infusion, with doses escalated in successive cohorts	Suspended	Duke University Medical Center, NC, United States	December, 2021
	Memory-enriched T cells in treating patients with recurrent or refractory grade III–IV glioma	1	42	NCT03389230	Memory-enriched T cells lentivirally transduced to express a HER2-specific, hinge-optimized, 41BB-costimulatory chimeric receptor and a truncated CD19	Recruiting	City of Hope Medical Center, CA, United States	December, 2023
EphA2	CAR-T cell immunotherapy for EphA2 positive malignant glioma patients	1 / 2	0	NCT02575261	Chimeric antigen receptor-modified T cells for EphA2	Withdrawn	Central Laboratory in Fuda Cancer Hospital, Guangdong, China	N/A
GD2	C7R-GD2.CAR T cells for patients with GD2-expressing brain tumors (GAIL-B)	1	34	NCT04099797	Intravenous injection of between 1 × 10^7^–1 × 10^8^ CAR-T cells with or without lymphodepletion chemotherapy	Recruiting	Baylor College of Medicine, TX, United States	February, 2023
CD147	CD147-CART cells in patients with recurrent malignant glioma	1	31	NCT04045847	Intracavity injection of CAR-T cells, once per week for three weeks	Recruiting	National Translational Science Center for Molecular Medicine & Department of Cell Biology Xi’an, Shaanxi, China	October, 2020
CAR T-cell therapy in combination with chemotherapy								
EGFRvIII	Immunogene-modified T (IgT) cells against glioblastoma multiforme	1	20	NCT03170141	Immunogene-modified Antigen-specific T (IgT) cells. Non-myeloablative chemotherapy consisting of fludarabine and/or cyclophosphamide, followed by intravenous infusion of autologous IgT cells. A standard 3 + 3 escalation approach will be used to obtain the safe dosage of IgT cells. The tested IgT cell dosage ranges from 0.5 × 10^5^/kg to 2.5 × 10^7^/kg	Enrolling by invitation	Shenzhen Geno-immune Medical Institute, Guangdong, China	December, 2022
CAR T-cell therapy in combination with immune check point inhibitors								
IL13Ra2	IL13Ralpha2-targeted chimeric antigen receptor (CAR) T cells with or without nivolumab and ipilimumab in treating patients with recurrent or refractory glioblastoma	1	60	NCT04003649	Intravenous administration of nivolumab and ipilimumab followed by intracranial-intraventricular/intracranial-intraventricular infusion of CAR-T cells. Up to four cycles	Recruiting	City of Hope Medical Center, CA, United States	December, 2022

Most recent GBM CAR-T cell therapy clinical trials were searched at www.Clinicaltrials.gov (2010 to present)

CAR-T cells have also been developed against the EGFRvIII antigen as mentioned above. The interest in the EGFRvIII variant stems from it being a mutated form of EGFR present in about 52% of glioma cells, but not healthy tissues [50]. In an EGFRvIII clinical study, the patients’ tumors also underwent antigenic loss, rendering the tumor CAR-T resistant [41]. It is interesting to note that despite knowledge regarding this phenomenon, it is not discouraging other groups from developing additional EGFRvIII-targeted CAR-Ts using novel higher affinity antibodies as recently as 2021 [51, 52]. Others have developed CAR-T secreting *Bi* specific *T* cell *E*ngagers or BiTEs targeting EGFRvIII [53].

On another front, in order to improve CAR-T control even further, recent work by Dr. Wendel Kim, building on the development of Syn-Notch receptors [54], has developed a sigmoidal Syn-Notch that can discriminate tumor antigens very accurately [55]. Even more recently, another take on Syn-Notch by their creators has been developed using a two-strike trivalent CAR-T against EGFRvIII and EphA2/IL-13Rα^+^ TanCAR in a GBM murine model [56]. However, Syn-Notch circuits are exogenous and could lead to allergic reactions, as mentioned in the original publication [57].

Several approaches have been developed to address the obstacle of antigenic loss (Fig. 2). Most notably, a novel CAR-T employing the scorpion toxin peptide Chlorotoxin (CLTX) has been developed recently to target CAR-Ts towards GBM antigens [58]. This CLTX-CAR-T induced long-term remission in murine studies [58]. Since CLTX is known to recognize 100% glioma cells through at least three tumor-associated proteins while not affecting healthy tissue [59], the clever rational behind this approach is that a CLTX-CAR-T could potentially reduce antigenic loss once and for all. However, through their very elegant set of studies, Wang et al., identified expression of the surface protein MMP-2 on glioma cells as necessary to mediate the CLTX binding. Loss of MMP2 in GBM -which has been described [60]- may hamper CLTX targeting. Current clinical trials are underway (NCT04214392) that will determine whether CLTX-CAR-Ts can improve GBM prognosis in patients. It is interesting to note that a CLTX-antibody was developed in 2012 but its clinical application remains unknown [61]. We have also compiled updated lists of current trials in Table 1 and completed trials in Table 2 adapted from reviews by Land C.A. et al. [62] and Maggs L. et al. [63]

**Table 2:** List of completed clinical trials involving CAR-T and GBM

Molecular target	CAR T-cell therapy	Phase	Estimated enrollment	Clinical trial reference number	CAR-T cell dosage	Location	Response
EGFRvIII	CAR T cell receptor immunotherapy targeting EGFRvIII for patients with malignant gliomas expressing EGFRvIII	1/2	18	NCT01454596	Two intravenous doses of 6.3 × 10^6^ to 2.6 × 10^10^ CAR-T cells per infusion, 2 h apart	National Institutes of Health Clinical Center, United States	Median survival 6.9 months Median progression-free survival 1.3 months, nil benefit (Goff et al. 2019)
	Autologous T cells redirected to EGFRVIII-with a chimeric antigen receptor in patients with EGFRVIII + glioblastoma	1	11	NCT02209376	Intravenous single dose of 1.75 × 10^8^–5 × 10^8^ CAR-T cells	Universtiy of Pennsylvania (University of California)	Median overall survival ~ 8 months, nil benefit Terminated (to pursue combination strategies) (O’Rourke et al. 2017)
	Pilot study of autologous anti-EGFRvIII CAR T Cells in recurrent glioblastoma multiforme	1	20	NCT02844062	CAR-T cells are infused intravenously to patients in a three-day split-dose regimen (day 0, 10%; day 1, 30%; day 2, 60%)with a total targeted dose	Sanbo Brain Hospital Capital Medical University, Beijing, China	Not reported
	CART-EGFRvIII + pembrolizumab in GBM	1	7	NCT03726515	CAR-T- EGFRvIII + Pembrolizumab	Universtiy of Pennsylvania	Not reported (terminated)
HER2	CMV-specific cytotoxic T lymphocytes expressing CAR targeting HER2 in patients with GBM	1	16	NCT01109095	One or more intravenous infusion of 1 × 10^6^/m^2^–1 × 10^8^/m^2^ CAR-T cells	Baylor College of Medicine, TX, United States	Median overall survival 24.5 months Median progression-free survival 3.5 months, 1 (6%) patient had partial response, 7 (44%) had stable disease (Ahmed et al. 2017)
IL13Ra2	Genetically modified T cells in treating patients with recurrent or refractory malignant glioma	1	92	NCT02208362	IL13Ra2-specific, hinge-optimized, 41BB/truncated CD19-expressing CAR-T cells by intratumoral, intracavitary, or intraventricular catheter Weekly for three weeks with additional infusions if eligible	City of Hope Medical Center, CA, United States	Not reported
	Cellular adoptive immunotherapy using genetically modified T-lymphocytes in treating patients with recurrent or refractory high-grade malignant glioma	1	3	NCT00730613	Intravenous infusions of up to 10^8^ CAR-T cells on days 1, 3 and 5 for 2 weeks. Treatment repeated after 3 weeks	City of Hope Medical Center, CA, United States National Cancer Center	Mean survival after relapse 11 months, positive response (Brown et al. 2016)
	Phase I study of cellular immunotherapy for recurrent/refractory malignant glioma using intratumoral infusions of GRm13Z40-2, an allogeneic CD8^+^ cytolitic T-cell line genetically modified to express the IL 13-zetakine and HyTK and to be resistant to glucocorticoids, in combination with interleukin-2	1	6	NCT01082926	Intratumoral infusions of GRm13Z40-2, an allogeneic CD8^+^ cytolitic T-cell line genetically modified to express the IL 13-zetakine and HyTK	City of Hope Medical Center, CA, United States	Median overall survival 19.7 months (Keu et al. 2017)
CAR T-cell therapy in combination with immune check point inhibitors							
EGFRvIII	CART-EGFRvIII + pembrolizumab in GBM	1	7	NCT03726515	EGFRvIII-directed CAR T cells combined with PD-1 inhibition (Keytruda)	Abramson Cancer Center of the University of Pennsylvania, PA, United States	Not reported
EGFRvIII, IL13Ra2, EphA2 or GD2	Personalized chimeric antigen receptor T cell immunotherapy for patients with recurrent malignant gliomas	1	100	NCT03423992	CAR-T cells expressing receptors specific for EGFRvIII, IL13Ra2, EphA2 or GD2, with or without anti-PD-L1 mAb	Xuanwu Hospital, Beijing, China	Not reported

Other CAR-T approaches to treat gliomas have been to include helper genes. As such, a recent advancement has been reported from Huang et al., who have engineered a CD70-CAR [64]. Over-expression of CD70 on glioma cells has been known to induce apoptosis on T-cells via CD27 [65] and Huang et al., cleverly replaced the intracellular portion of CD27 by 41BB and CD3ζ, thus basically turning CD27 into a CD70 targeting CAR-T. The authors showed that this type of CAR could kill CD70^+^ glioma cells in vitro and in vivo, although remission was only achieved using 100 × 10^6^ CAR-T cells [64]. Then, in a follow-up study, they co-expressed CXCR2 expression using a Furin-V5-P2A sequence [66] with their CD70-CAR in order to guide the modified T-cells towards IL-8 producing glioma cells [67]. Since IL-8 expression is known to promote tumor resistance and invasion [68] besides being one of the predominantly expressed chemokines in GBM [69], the authors rationale is that it would help T-cells hone in on, and infiltrate tumors more efficiently. Using this approach, the authors needed only 2 × 10^6^ CARs to achieve remission in vivo in preclinical modeling after 150 days [67]. Interestingly, since the authors were able to treat not just U87 murine xenographs but also pancreatic and ovarian-type cell lines, it seems co-expression of CXCR2 could improve treatment of solid tumors overall. However, it is unknown whether CD70^+^ glioma cells can undergo CD70 loss under long-term CAR-T pressure, so reproducibility and clinical feasibility of these studies needs further investigation. However, CD70 CAR-T clinical trials involving solid tumors such as pancreatic, renal and breast cancer were recently suspended after enrolling only two patients (NCT02830724). CD70 has also been combined with targeting B7-H3 (CD276) with a TanCAR strategy (Fig. 2G) that showed enhanced anti-tumor functionality against gliomas and many other solid tumors [70].

Another helper gene recently used has been IL-15 [71]. IL-15 is a cytokine that has been demonstrated to enhance survival of T-cells. However, use of this cytokine in vivo is complex, since it needs to be expressed in trans with its receptor in order to extend IL-15 half-life and thus function effectively in vivo [72].

In order to address the glioma antigenic escape problem, a daring Trivalent CAR strategy was developed by Bielamowicz et al. [73], using a single lentiviral construct expressing three individual CARs directed against IL-13Rα2, HER and EphA2. The authors reported almost 100% success using two different patient-derived cell line xenographs in five mice during a 60-day period. However, several limitations appear to surround this approach. Requirements for producing high enough viral titers for such long constructs in a clinical setting remains to be seen [74] and the inter- and intra-patient GBM variability appears to be wider than what the authors describe a Universal CAR or UCAR can achieve [75]. However, if such strategy can be proven effective at containing the growth of GBM, surely the manufacturing of such large constructs would find a way to translate clinically.

Owing to the versatility that transmembrane proteins confer, several other types and combinations of CARs have been developed and are listed in Supplementary Table 1, however, their viability and utilization against GBM remains to be seen.

However, solving the challenge of antigenic loss may not be the only obstacle in order to achieve long-term remission of gliomas. The TME and the alleged detrimental effects it exerts on T-cells such as T-cell exhaustion and poor tumor trafficking, are major obstacles that need to be account for in any new strategies [76–78]. As Wang et al., suggest in their studies, some CLTX-CARs that failed to achieve durable remission in murine models may be due to T-cell exhaustion [58].

### Combined CAR-T use with other therapies

Recently, CAR-T therapeutics aimed at GBM have been used in combination with existing anti-tumor agents, such as temozolamide [79] or immune checkpoint inhibitors (ICI) PD1/PD-L1 against refractory diffuse large B-cell lymphoma [80]. However, the clinical efficacy of ICI against GBM may be moot with open questions regarding their usefulness against GBM [81] after usage of Nivolumab –a PD1/PD-L1 inhibitor- failed to show any clinical benefit to patients with recurrent GBM [82]. Owing to the plastic nature of CAR-Ts, work has also been published using CAR-Ts of many carriers of oncolytic viruses [83] or oncolytic viruses targeting IL-13Rα2 [84]. Additionally, modified CAR-NK cells have been used to eliminate MDSC before CAR-T administration in murine models [85] as well as co-expressing CXCR4 and EGFRvIII in NK cells to improve immunotherapy against CXCL12-secreteing GBM [86].

### Special details of CAR-T modules

Since the development of additional co-stimulatory modules to CAR-Ts in order to improve their in vivo potency and lasting effect (Fig. 1B), several additional modifications to certain modules have been reported or are de facto known and used in the CAR-T community. One of these is a key modification on the CD28 co-stimulatory domain where a single amino acid change involving an asparagine to a phenylalanine (N193F) improves long-term survival and exhaustion of T-cells [87]. Interesting too is the recently reported non-canonical CD3e motif -RKxQRxxY- that has also been described to provide improved function [88]. Improvements on the stem/hinge section of CAR-Ts have also been reported. For example, a mutant with two amino acid changes -L235E and N297Q- thus coined EQ on the IgG4 domain is reported to improve T cell persistence and anti-tumor efficacy while avoiding Fc receptor binding in CD19-CAR-Ts [89]. One should also note that several modifications of co-stimulatory domains or hinges are not reported in the literature, but are described in filed patents, such as the change of two leucines to glycines in the CD28 intracellular domain (L186G and L187G) designated LLmGG [90] and recent patent WO2017066481A1. Schönfeld et al., have also filed a patent reporting that a serine to cysteine change in the CD8 hinge regions (S164C) improves functionality and expression of CAR-T [91] and patent EP3115373A1. Finally, a double mutant CD3ζ domain with mutation Q65K and deletion Q101 has been reported from Mackall’s group, albeit no functional difference from the wt sequence was noted (Dr. Benjamin Salzer, personal communication and present in patent WO/2020/118076).

### Untested approaches in gliomas

Several novel therapeutic approaches involving T cell engineering have been developed, but none have been tested against gliomas or GBM. For example, CD40 is known to be upregulated in 40% of all GBM [92] and constitutive expression of its ligand CD40L on CAR-T has been shown to enhance IL-12 secretion, extend survival of T cells, and increase cytotoxicity against tumors [93]. Other groups have engineered heat-controlled CAR-T that display enhanced intra-tumoral activity [94].

Last year a novel monomorphic MHC class I-related protein, MR1, was described to be expressed in virtually all types of cancers but not healthy cells [95]. Authors showed this particular surface MR-1 molecule could be targeted by a TCR that recognizes vitamin B-related metabolites present in malignant cells but not healthy ones. Although MR1-restricted mRNA has been detected in glioma cell lines such as U-373 [96], its expression in another commonly used glioma cell line U87 or patient-derived GSC is unknown. Another promising approach has been the development of Supra-CARs, i.e., inter-changeable CAR-Ts that can be modulated by soluble adaptors [97]. Protein logic, i.e., combinatorial antigens- targeting HER2^+^ cells, have also been developed to achieve precise discriminatory effector functions targeting antigens present in tumor, but not healthy, cells [98].

Another promising approach to developing CARs targeting difficult tumor antigens was used by Rafiq et al., in 2017, creating a CAR with the ability to target intracellular Tumor-Associated Antigens (TAA) such as Wilms tumor 1 (WT1), known to be up-regulated in gliomas [99]. Using this approach, the CAR is guided by an antibody portion that recognizes a surface MHC molecule loaded with peptide fragments of WT1 [99], which otherwise cannot be accessible to T-cells as it is ordinarily intracellular (Fig. 2I). WT1 is an oncogenic, zinc-finger transcription factor involved in differentiation, proliferation and apoptosis among other functions [100]. WT1 is known to be over-expressed in many malignancies, including GBM [101], offering broad tumor therapeutic potential. Expanding on this strategy to target MHC-presented peptides from key intracellular oncoproteins, Yamarkovich M. et al. have recently created a peptide-centric CAR (PC-CAR) that can target neuroblastoma dependency gene and intracellular transcriptional regulator PHOX2B and induce remission in a murine model of neuroblastoma [102].

In order to increase local potency of CAR-Ts, Fourth Generation CAR-Ts—“T cells redirected for antigen-unrestricted cytokine-initiated killing” (TRUCKS) have been tested in murine models [103]. As shown in Fig. 2, CAR-T along with NFAT-controlled cytokines IL-12 or IL-18 can increase potency against tumors only after the primary CAR-T is engaged and T cells activate [104, 105]. However, although IL-12 TRUCKs showed efficacy in murine models, they provided little therapeutic effects in a Phase 1 clinical trial for metastatic seminal vesicle cancer, although no adverse side effects were reported [106]. An additional detrimental effect of this approach is that NFAT-controlled cytokines might activate independently of the primary CAR-T engagement if the T-cell is activated by alternative mechanisms. This hindrance may be solved using Syn-Notch receptors as introduced above. Other cytokines that have been constitutively co-expressed along with CAR-Ts include IL-7 and CLL19, which promote survival via decreased T-cell exhaustion- and tumor infiltration respectively [107]. More recently, CAR-Ts constitutively expressing IL-9 have also been described as having a superior tumor-fighting phenotype against liquid and solid tumors, displaying central memory phenotype, decreased exhaustion markers and robust proliferative capacity [108].

Delivering the gene cargo into CAR-Ts has also been a logistical problem, and its difficulty in clinical translation of bench developments have been mentioned. Buchholz et al., have developed a lentivirus capable of creating CD19-CAR-T cells in vivo without the requirement for cell expansion [109]. Utilization of this approach versus solid tumors remains to be explored.

Many CAR-T improvements have been developed using combinatorial antigens [110] or AvidCARs [57] to improve selective tumor eradication. It has also been demonstrated that it is possible to restrict CAR-T expression to anoxic conditions similar to the hypoxic environment, a hallmark of GBM, by C-terminally attaching a HIF motif into the CAR that grants it oxygen-sensing features [111]. Scientists have also developed adaptor molecules that can recognize specific antigens while engaging the TCR machinery, thus minimizing tonic signaling [112]. One such approach is called T cell antigen coupler or TAC and has demonstrated efficacy in murine models against HER^+^ cells [113], another known surface antigen over-expressed in GBM. Other approaches have been even more daring such as directly combining antibody-based targeting domains with the TCR-like activation machinery itself [114]. In this approach, Yue Liu et al., showed that synthetic *T*CR and antigen receptor or STAR receptors, offer greater functionality compared to conventional CAR-Ts.

However, besides the clinical feasibility of these groundbreaking studies, a major obstacle that plagues most CAR-T or T cell engineering studies is the virtual impossibility to exactly reproduce CAR-T or similar expression experiments. Most often published reports do not include the exact protein sequence of the constructs used, or they do so in a convoluted manner that makes reproducibility very difficult and time consuming. Obtaining the original plasmids used by the authors from which the constructs are expressed is even more difficult—even though it is encouraged in published work. Sometimes, the only recourse is to reverse engineer the constructs or obtain the sequences from other source documentation such as patent applications. This adds another level of ambiguity, where one can never be completely sure whether the construct is the actual one used or not in the published work. This observation is compounded by the inflexible nature of proteins, where a single amino acid change can be very influential. In order to help readers regarding this issue, we have compiled a list in Fig. 3 and accompanying Supplementary Table 1 describing the constructs used in each study for which we were able to find the precise amino acid sequences. Hopefully, this will aid researchers in dissemination of vital information necessary to propel research forward and advance potential treatments for this devastating disease.

### CAR safety, efficacy and caveats in GBM

Safety concerns are a major issue when using CAR-Ts [115]. Targeting tumor cells while ensuring normal tissue remains undamaged needs to be extensively assessed using in vitro and preclinical in vivo testing prior to testing their clinical efficacy in human patients. One of the main toxicities associated with CAR-T therapy is induction of cytokine release syndrome (CRS) as well as poorly understood neurological sequelae. Fortunately CRS can be managed with the use of anti-IL-6 [116] and corticosteroids, but it can reduce the benefits of CAR-T as a result. For example, it was recently mentioned in an online conference-CelliCon Valley 2021- by Dr. Carl June, that a subgroup of neurons express very low levels of CD19 which might make them a target for the CD19-CAR-T approved clinically. However, the physiodynamics underlying CRS are extensive and could well fill a separate review [25, 115, 117–119].

Since existing patient T-cells need to be engineered in order to express the CAR-T, isolation, extraction and expansion of functional peripheral T-cells in a Glioma patient is required. This can be a challenge itself as GBM patients are heavily immunosuppressed and their T-cells are documented to have a wide range of T-cell dysfunction including, senescence, anergy, tolerance and exhaustion [120]. On top of this, the GBM median survival of 15–17 months after diagnosis can limit the timing of isolation, expansion and re-infusion of modified T-cells back into patients, since production alone of CAR-T can generally take approximately 2–4 weeks or more depending on the patient’s clinical status and chemotherapy usage [121, 122]. In order to shorten this step, off-the-shelf CAR-T are under investigation but their clinical usefulness has not been assessed yet [123].

## Conclusions

GBM tumors are among the most devastating types of cancers to afflict humans. As such, it is highly unlikely any single conventional or unconventional treatment that worked against other tumor types will decisively reverse unstoppable growth or adaptability and escape of GBM tumors. In this regard, CAR-T therapies have proven to be no exception despite their decisive role in reversing the prognosis of patients with liquid tumor malignancies. However, the advantage CAR-Ts offer versus other therapies is the unlimited potential for improvement via refined T cell engineering. Taking advantage of the flexible nature of DNA and delivery systems, it is possible that effective CAR-T combinations versus solid tumors such as GBM will be developed in the foreseeable future. In order to achieve this goal, a holistic approach should be undertaken. One in which several immunologic obstacles are addressed simultaneously including the challenge of antigenic loss, protecting CAR-T from the TME, T cell exhaustion, improving tumor homing, and making T cells resistant to immunosuppression. There is also room for improvement on safety profiles in order to reduce cytokine release syndrome, neurotoxicity and long-term control through iCasp9 [124], HSV-TK [125] gene or a suicide epitope [126]. However, the future is very promising, with novel and very innovative improvements being reported almost weekly. As such, the possibilities of CAR-T and T cell engineering has never looked more promising in designing potential therapeutics for treatment of solid tumors and specifically GBM, the meanest of them all.

## Supplementary Information

Below is the link to the electronic supplementary material.

Supplementary Table 1 (XLSX 21 kb)

## Data Availability

Data sharing not applicable to this article as no datasets were generated or analyzed during the current study.
